# Reduced Graphene-Oxide-Encapsulated MoS_2_/Carbon Nanofiber Composite Electrode for High-Performance Na-Ion Batteries

**DOI:** 10.3390/nano11102691

**Published:** 2021-10-13

**Authors:** Su-Ho Cho, Jong-Heon Kim, Il-Gyu Kim, Jeong-Ho Park, Ji-Won Jung, Hyun-Suk Kim, Il-Doo Kim

**Affiliations:** 1 Department of Materials Science and Engineering, Korea Advanced Institute of Science and Technology (KAIST), Daejeon 34141, Korea; caca1108@kaist.ac.kr; 2 Department of Materials Science and Engineering, Chungnam National University, Daejeon 34134, Korea; mapig2@naver.com; 3 School of Materials Science and Engineering, University of Ulsan, Ulsan 44776, Korea; ilgyu.kim.96@gmail.com (I.-G.K.); fjdk9936@naver.com (J.-H.P.)

**Keywords:** molybdenum sulfides, carbon nanofibers, reduced graphene oxides, anodes, sodium-ion batteries

## Abstract

Sodium-ion batteries (SIBs) have been increasingly studied due to sodium (Na) being an inexpensive ionic resource (Na) and their battery chemistry being similar to that of current lithium-ion batteries (LIBs). However, SIBs have faced substantial challenges in developing high-performance anode materials that can reversibly store Na^+^ in the host structure. To address these challenges, molybdenum sulfide (MoS_2_)-based active materials have been considered as promising anodes, owing to the two-dimensional layered structure of MoS_2_ for stably (de)inserting Na^+^. Nevertheless, intrinsic issues of MoS_2_—such as low electronic conductivity and the loss of active S elements after a conversion reaction—have limited the viability of MoS_2_ in practical SIBs. Here, we report MoS_2_ embedded in carbon nanofibers encapsulated with a reduced graphene oxide (MoS_2_@CNFs@rGO) composite for SIB anodes. The MoS_2_@CNFs@rGO delivered a high capacity of 345.8 mAh g^−1^ at a current density of 100 mA g^−1^ for 90 cycles. The CNFs and rGO were synergistically taken into account for providing rapid pathways for electrons and preventing the dissolution of S sources during repetitive conversion reactions. This work offers a new point of view to realize MoS_2_-based anode materials in practical SIBs.

## 1. Introduction

A battery of things (BoT)—first mentioned by Tony Seba, the author of “Clean Disruption of Energy and Transportation”—has come for modern society, requiring battery-powered devices everywhere [1]. Current lithium-ion batteries (LIBs), with a high energy density of ~250 Wh kg^−1^, have been considered to be promising energy storage systems combined with renewable energy technologies [2]. Despite the viability of LIBs, utilization of the state-of-the-art LIBs for an energy storage system (ESS) has been restricted due to finite reserves of Li^+^ on earth, which would increase the price of Li sources used for large-scale applications [3]. Among alternatives to LIBs, sodium-ion batteries (SIBs) with a similar battery chemistry (redox potential of −2.71 V vs. SHE) as LIBs have constituted an exciting avenue for advancing ESSs [4,5].

However, anode materials with low energy density have impeded the development of SIB technologies. To address this issue, many promising candidates (e.g., hard carbon, metal oxides/sulfides/selenides, etc.) with high capacities have been suggested so far [6,7,8,9,10,11,12]. However, carbonaceous materials show limited use due to their low specific capacity and less reversible capacity, and transition metal oxides (TMOs) need to be changed due to their low conductivity. As high-capacity anodes, sulfide-based materials such as FeS_2_ and Sn_2_S have been developed because transition metal sulfides (TMSs) have a higher electronic conductivity as well as an excellent ability to store Na^+^ [11,12]. Among them, molybdenum sulfide (MoS_2_)-based anode materials possessing large interlayer spaces (0.615 nm) between two-dimensional (2D) MoS_2_ slabs could allow facile (de)insertion of Na^+^ with few structural changes during conversion reactions (MoS_2_ + 4Na^+^ + 4e^−^ → Mo_(metallic)_ + 2Na_2_S, a theoretical capacity of 670 mAh g^−1^), leading to great electrochemical performance [13,14]. However, MoS_2_ has an intrinsically low electronic conductivity, lowering the efficiency of (dis) charging SIB cells [15,16]. In addition, long channels of 2D MoS_2_ interlayers are the bottleneck to Na^+^ diffusion, degrading SIB performance toward Na^+^ storage [17,18]. Furthermore, the dissolution of sulfur atoms contributes to a large loss in the overall mass after the conversion reactions, which is detrimental to achieving excellent battery performance. Therefore, we need to find a strategy to overcome the problems of MoS_2_ mentioned above [19,20,21].

In this work, we successfully fabricated interlayer-enlarged MoS_2_ nanoflakes, which were doubly covered with carbon nanofibers (CNFs), and reduced graphene oxide (rGO) (MoS_2_@CNFs@rGO). The MoS_2_ nanoflakes were first confined in the CNFs by thermolysis, subsequently encapsulated by the rGO via electrostatic interaction and reduction processes. The interlayers of MoS_2_ nanoflakes in the CNFs were expanded during the thermolysis at 800 °C under the H_2_ atmosphere. Moreover, the synergy of CNFs and rGO not only increase the electronic conductivity of the composite but also prevent the loss of S, enabling the SIB cells to be operated reversibly with a high capacity of 345.8 mA g^−1^ for 90 cycles at a high current density of 100 mA g^−1^. With the support of experimental and analytical studies, the underlying reaction mechanism of MoS_2_@CNFs@rGO was investigated and proposed.

## 2. Materials and Methods

### 2.1. Experimental

Firstly, the MoS_2_@CNFs were synthesized by electrospinning and a thermolysis process [17]. For the electrospinning, a solution containing 15 wt % of ammonium tetrathiomolybdate ((NH_4_)_2_MoS_4_, ATTM, Alfa Aesar, Ward Hill, MA, USA) and 15 wt % of poly(styrene-acrylonitrile, SAN, Mw = 1,300,000) dissolved in 10 mL of *N*,*N*-dimethylformamide (DMF, Sigma-Aldrich, Burlington, MA, USA), was prepared on the hot plate by stirring the solution at 70 °C for 12 h. The solution was electrospun by applying a high voltage of 15 kV using an electrospinning machine (NanoNC, Seoul, South Korea). After the electrospinning, the electrospun NFs were thermally treated under H_2_/Ar (4/96, *v*/*v*) surroundings at 450 °C for 2 h and under a pure Ar (99.999%) atmosphere at 800 °C for 6 h, respectively [17,22]. These processes were performed to make the MoS_2_@CNFs. For wrapping the entire surfaces of MoS_2_@rGO with the rGO, the Ti-O-Ti-O atomic layers (sub-nm) were coated on the MoS_2_@rGO by using atomic layer deposition (ALD); this forms hydroxyl groups (OH-) of the atomic layers on MoS_2_@rGO for rGO wrapping [7,23]. Then, poly(allylamine hydrochloride, Mw = 900,000, Sigma-Aldrich) was utilized as a surface modifier to form an amine group (NH_2_-) on the surface, inducing a positively charged surface, i.e., -NH^3+^-grafted MoS_2_@CNFs, in an aqueous solution. The MoS_2_@rGO was added to the PAH solution. After stirring for 2 h, the PAH-modified MoS_2_@rGO was rinsed three times with distilled water and dried at 60 °C in a vacuum oven overnight. Lastly, the modified sample was encapsulated with rGO according to the method in the same manner of our previous works [7,24], resulting in MoS_2_@CNFs@rGO.

### 2.2. Materials Characterization

Nova 230 (field-emission scanning electron microscope (FE-SEM), FEI, Hillsboro, OR, USA) was employed to obtain FE-SEM images. The crystal structure of MoS_2_@CNFs@rGO was investigated by X-ray diffraction (XRD) patterns using D/Max-2500, with RIGAKU Corp. (Tokyo, Japan) with Cu Kα (λ = 1.54 Å) between 10° and 80° at a scan rate of 0.066° s^−1^. Both internal and external morphologies of MoS_2_@CNFs@rGO and the distribution of elementals were analyzed by a high-resolution transmission electron microscope (HR-TEM) operating at 300 kV and a scanning TEM (STEM) using a Tecnai F30 S-Twin (FEI, Hillsboro, OR, USA) equipped with energy-dispersive X-ray spectroscopy (EDX). The chemical states of MoS_2_@CNFs@rGO were investigated by X-ray photoelectron spectroscopy (XPS, K-alpha, Thermo VG Scientific, Waltham, MA, USA). In addition, the dominant vibration modes in the MoS_2_@CNFs@rGO were investigated using Raman spectroscopy (ARAMIS, Horiba Jobin Yvon, Montpellier, France) with a 514 nm laser source.

### 2.3. Electrochemical Evaluation

All the electrodes were prepared by casting a slurry, including active materials (80%), a conducting agent (Super-P, Sigma-Aldrich, Burlington, MA, USA) (10%), and a polyvinylidene fluoride (PVDF, Mw ~534,000, Sigma-Aldrich, Burlington, MA, USA) binder (10%); the slurry was mixed together using an agate mortar and cast on a copper foil as a current collector. After casting, the electrode was dried in a vacuum oven for 12 h. The mass loading of the MoS_2_@CNFs@rGO was approximately 1.0 ± 0.1 mg cm^−2^. Half-cell (2032 type-coin cell) assembly was done in an Ar-filled glovebox (water content < 0.1 ppm). Na metal was used as a counter electrode, and a Whatman glass microfilter was employed as a separator for SIB cell tests. The used SIB electrolyte was 1 M NaClO_4_ in propylene carbonate (PC) with 5 wt % of FEC. The assembled coin cells for Na storge were cycled at a current density of 100~20,000 mA g^−^^1^ between 0.005 and 3.0 V using a battery tester (WBCS3000 device by WonATech, Seoul, South Korea).

## 3. Results

### 3.1. Materials Characterization

**Figure 1:**
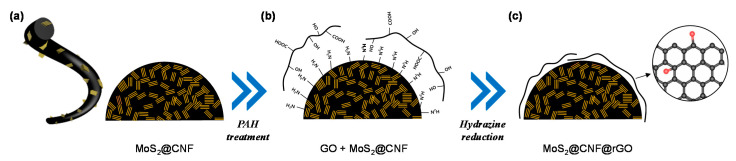
Schematic illustration for preparation of MoS_2_@CNFs@rGO. (**a**) Fabrication of MoS_2_@CNFs synthesized by thermolysis after electrospinning. (**b**) Electrostatic interaction between PAH-modified MoS_2_@CNFs and GO. (**c**) The final product of rGO-wrapped MoS_2_@CNFs achieved after hydrazine reduction.

The synthetic procedures to prepare the MoS_2_ nanoflakes confined in CNFs wrapped with the rGO net (MoS_2_@CNFs@rGO) are schematically illustrated in Figure 1. The MoS_2_-nanoflake-embedded carbon nanofibers (MoS_2_@CNFs) were prepared by thermal treatment using ammonium tetrathiomolybdate ((NH_4_)_2_MoS_4_) and poly(styrene-acrylonitrile) (SAN) as precursors through the electrospinning method (Figure 1a). First, the (NH_4_)_2_MoS_4_ was decomposed into MoS_2_ at 450 °C in the presence of reducing (H_2_) gas [25,26]. Then, carbonization and crystallization steps for MoS_2_ are performed through heat treatment at 800 °C under an inert gas environment. The reactions that occur during the heat treatment process are as follows.

(1) $${\left({\mathrm{NH}}_{4}\right)}_{2}{\mathrm{MoS}}_{4}\;\to {\mathrm{MoS}}_{3}+2{\mathrm{NH}}_{3}↑+{\;H}_{2}S↑$$

(2) $${\mathrm{MoS}}_{3}\;\to {\;\mathrm{MoS}}_{2}+\;S$$

(3) $${\left({\mathrm{NH}}_{4}\right)}_{2}{\mathrm{MoS}}_{4}+H_{2}\to {\mathrm{MoS}}_{2}+2{\mathrm{NH}}_{3}↑+2H_{2}S↑$$

For rGO coating on the MoS_2_@CNFs, the surface of MoS_2_@CNFs was modified using poly(allylamine hydrochloride) (PAH) [7,23,24]. Firstly, the MoS_2_@CNFs were dipped in PAH solution; the surface functional group changed from the hydroxy surface group to the amine group (-NH_2_) (see the details in the Materials and Methods section). The PAH-modified MoS_2_@CNFs have positively charged amine functional groups (NH_3_^+^) exposed on the surface. On the other hand, the GO has functional groups of the carboxyl group (-COOH) and hydroxy group (-OH), attributed to the negative charge of GO in the aqueous solution. The GO flakes were quickly attracted to the surface of PAH-modified MoS_2_@CNFs due to the electrostatic self-assembly (Figure 1b). Then, the GO was chemically reduced by hydrazine treatment, which rendered the strong chemical bond between the amine functional group and oxygen group with the ring-opening reaction. Finally, the GO changed to rGO, covering the entire surface of MoS_2_@CNFs (Figure 1c).

**Figure 2:**
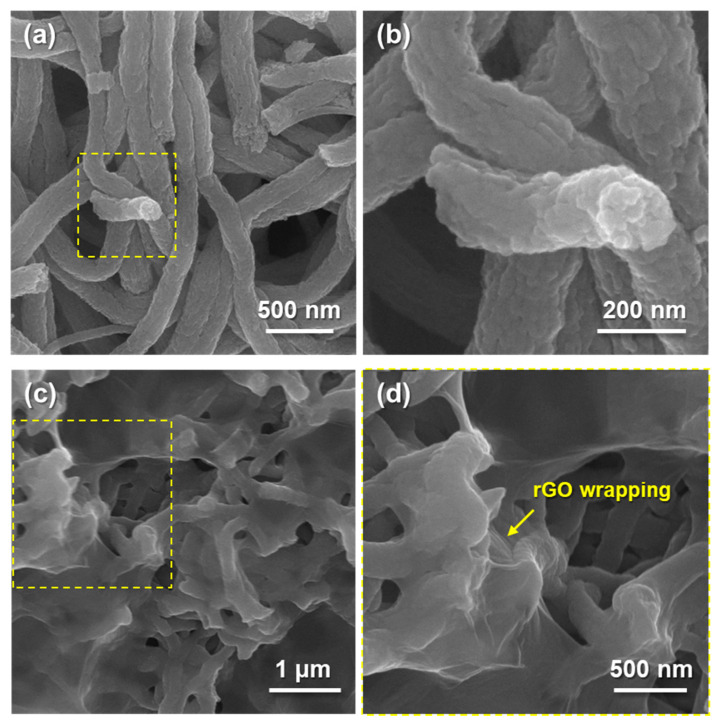
SEM images of (**a**) MoS_2_@CNFs and (**b**) MoS_2_@CNFs@rGO, HR-SEM images of (**c**) MoS_2_@CNFs and (**d**) MoS_2_@CNFs@rGO.

**Figure 3:**
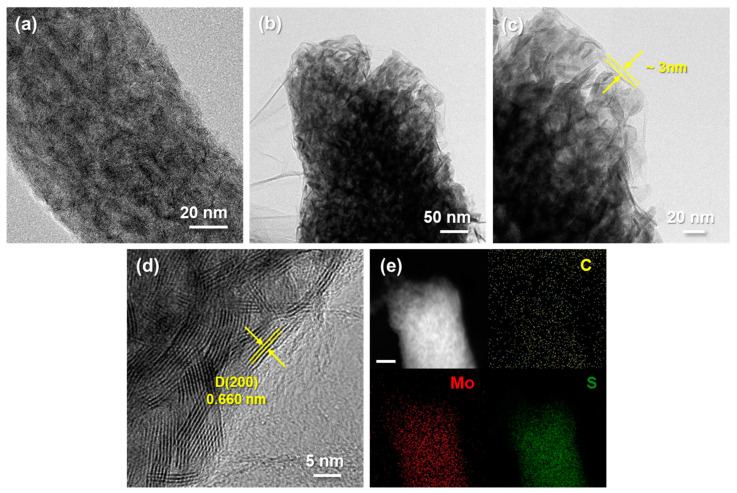
TEM images of (**a**) MoS_2_@CNFs and (**b**) MoS_2_@CNFs@rGO, (**c**,**d**) HR-TEM images of MoS_2_@CNFs@rGO, (**e**) STEM image and elemental distribution of C, Mo, and S in the MoS_2_@CNFs@rGO (scale bar: 20 nm).

The morphology of synthesized MoS_2_@CNFs is shown in Figure 2a,b, demonstrating that the nanocomposite possessed one-dimensional (1D) NF networks, with the diameter of each fiber at about 200 nm and several microsized pores among the NFs. These pores facilitate electrolyte penetration to enhance electrochemical performance. The morphological change of MoS_2_@CNFs@rGO was confirmed through SEM images (Figure 2c,d). It is confirmed that rGO flakes were covered on the NF surfaces while the 1D architecture of MoS_2_@CNFs was maintained. To verify the internal structure and phase of the MoS_2_@CNFs and MoS_2_@CNFs@rGO, transmission electron microscopy (TEM) analysis was conducted. For the MoS_2_@CNFs, few-layer MoS_2_ flakes were uniformly distributed into CNFs (Figure 3a).

After the graphene wrapping process, the rGO flakes substantially covered the MoS_2_@CNFs without any aggregations. Individual NFs were wrapped by stacked rGO flakes with a thickness of approximately 3 nm (Figure 3b,c). We expected that the favorable influx of Na^+^ into MoS_2_@CNFs would be possible through the thin rGO layer, and the electrically conductive rGO would enhance the electrical efficiency of the battery cell by facilitating the electron transport. Figure 3d shows a high-resolution TEM (HR-TEM) image of MoS_2_@CNFs@rGO. The lattice fringe of the MoS_2_ in the MoS_2_@CNFs@rGO is approximately 0.660 nm, indicating that the multilayer MoS_2_ nanoflakes had enlarged interlayer spacing compared to the general interlayer spacing (~0.615 nm) of multilayer MoS_2_ [27,28]. The enlarged interlayers can be attributed to the CNFs suppressing the crystallization of MoS_2_ during thermolysis. In addition, the wide interlayer distance might allow Na^+^ to diffuse rapidly, promoting Na^+^ flux in the whole electrode. The homogeneous distribution of elements—such as C (yellow), Mo (red), and S (green)—in the MoS_2_@CNFs@rGO was investigated by energy-dispersive spectroscopy (EDS) mapping images from a scanning TEM (Figure 3e). The elements were well-distributed in the 1D NF composite, which can be supported by the qualitative data in Figure S1.

**Figure 4:**
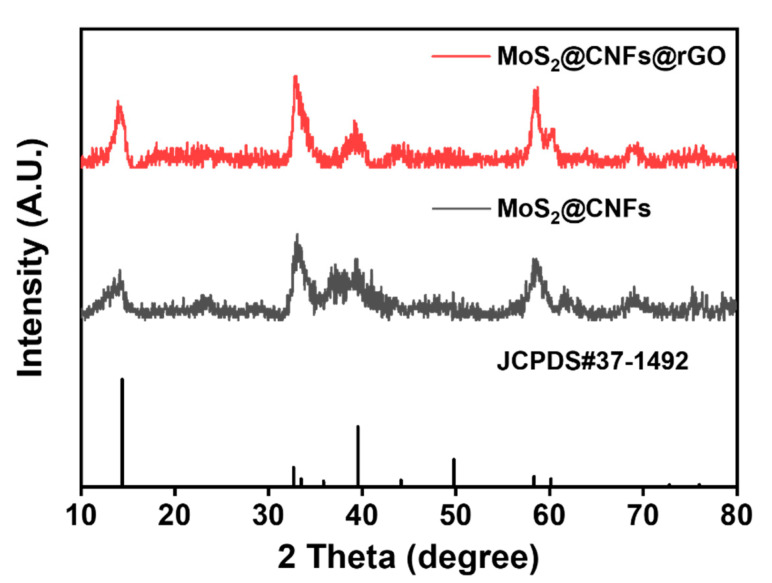
X-ray diffraction pattern of MoS_2_@CNFs and MoS_2_@CNFs@rGO.

**Figure 5:**
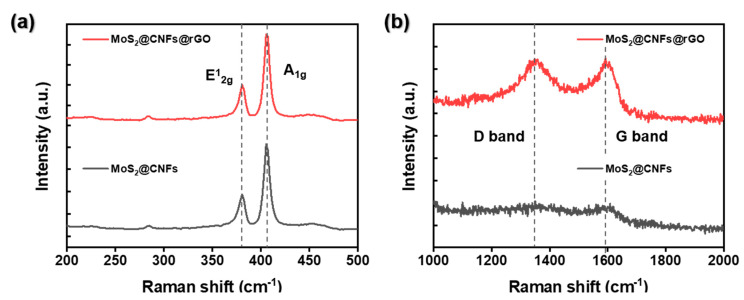
Results of Raman analyses of MoS_2_@CNFs and MoS_2_@CNFs@rGO with different ranges: (**a**) 200~500 cm^−1^, (**b**) 1000~2000 cm^−1^.

To further consider the crystal structure and phase information of the MoS_2_@CNFs@rGO, Figure 4 exhibits the X-ray diffraction (XRD) patterns of MoS_2_@CNFs and MoS_2_@CNFs@rGO. At 14.2°, 33.3°, and 59.1°, the XRD peaks correspond to planes (002), (100), and (110) of the MoS_2_ phase (JCPDS #37-1492), respectively [29]. Through the graphene wrapping process, no peak shifts were observed for MoS_2_@CNFs@rGO. Furthermore, as a result of XPS analysis, the chemical states of Mo and C of MoS_2_@CNFs@rGO showed insignificant change despite the chemical reduction reaction. (Figure S2). Figure 5 compares the Raman spectra of MoS_2_@CNFs and MoS_2_@CNFs and shows the vibrational modes, the fingerprint of the chemical state of the MoS_2_ phase. MoS_2_@CNFs and MoS_2_@CNFs@rGO represent two peaks at 379.1 and 402.7 cm^−1^ due to in-plane E^1^_2g_ and out-of-plane A_1g_ vibration modes [30]. Several studies exhibited that the relative intensity of these two peaks suggests the characteristics of MoS_2_ crystals, given dimensions and edge profiles. Generally, the intensity of the A_1g_ mode is greater than that of the E^1^_2g_ mode when the MoS_2_ flakes have an edge-end structure. There is no difference between the E^1^_2g_ and A_1g_ mode peaks for MoS_2_@CNFs and MoS_2_@CNFs@rGO, indicating that the MoS_2_ nanoflakes in the CNFs maintain their structures without damage during the fabrication process. Furthermore, introducing the rGO layer increases the intensity of the D and G bands, which indicates the carbon structure (Figure 5b and Figure S3) [31,32]. The integrated area ratio of sp^3^ to sp^2^ (Asp^3^/Asp^2^) has been proven to provide useful information concerning the nature of carbon [32]. The low ratio of Asp^3^/Asp^2^ indicates the presence of a large amount of sp^2^ carbon. The Asp^3^/Asp^2^ were 1.31 for MoS_2_@CNFs and 1.15 for MoS_2_@CNFs@rGO, respectively. It means that the amount of sp^2^-type carbon increased after rGO wrapping and the reduced graphene oxide layers are well-introduced in the MoS_2_@CNFs@rGO sample.

To quantitatively identify the contents of rGO in MoS_2_@CNFs@rGO, element analysis (EA) was carried out (Table 1). The MoS_2_@CNFs contain 23.6 wt % of carbon and about 29.8 wt % of sulfur from MoS_2_. After graphene wrapping, the amount of carbon (C) increased to 34.3 wt %, indicating that the sulfur (S) content was relatively decreased. Assuming that the ratio of CNF/MoS_2_ is maintained after graphene wrapping, it is confirmed that the content of rGO accounts for about 17.7 wt % in the MoS_2_@CNFs@rGO. Since the 1D nanostructure of MoS_2_@CNFs has a large surface area, a sufficient amount of rGO is required for the wrapping, even if it contains thin rGO layers. Therefore, the rGO should cover both the individual NFs and bundle of MoS_2_@CNFs, increasing the electronic conductivity of the MoS_2_@CNFs@rGO and preserving active materials, particularly S, during repetitive reactions [33,34].

### 3.2. Electrochemical Measerment

**Figure 6:**
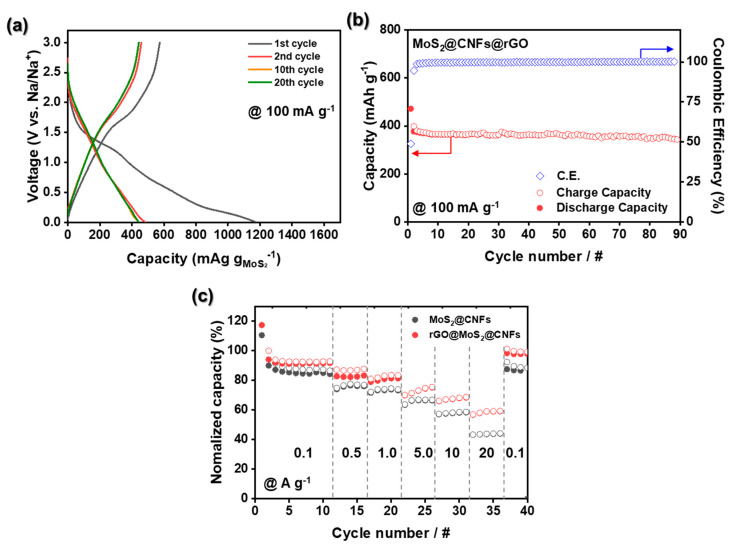
Electrochemical performances of MoS_2_@CNFs@rGO. (**a**) Charge–discharge profile at 100 mA g^−1^ between 0.005 V~3.0 V vs. Na/Na^+^. (**b**) Cycling performance at 100 mA g^−1^. (**c**) Rate capability with different current densities (0.1~20 A g^−1^).

In general, MoS_2_ electrochemically reacts with Na^+^ based on insertion and conversion reactions [35]. To testify to these electrochemical behaviors in the MoS_2_@CNFs@rGO, the galvanostatic charge-discharge curves for the MoS_2_@CNFs@rGO were obtained in a voltage window between 0.005~3.0 V (vs. Na/Na^+^) at a current density of 100 mA g^−1^ (Figure 6a). The initial discharge and charge capacities of MoS_2_@CNFs@rGO are 1175 and 573 mAh g^−1^, respectively, corresponding to a Coulombic efficiency (CE) of 48%. This capacity fading for the initial cycle is attributed to the generation of high irreversible capacitance stemming from the solid-electrolyte interphase (SEI) layers formed on the electrode with a large surface area. Nevertheless, in Figure S4, the initial CE of MoS_2_@CNFs@rGO is higher than that of MoS_2_@CNFs (44%). It appears that more SEI layers seem to form on the MoS_2_@CNFs without the rGO coating layers while the rGO adequately stabilizes the SEI layers on the MoS_2_@CNFs@rGO [36]. Moreover, rGO played a critical role in accelerating electron transport, which improved the initial CE of MoS_2_@CNFs@rGO. It is confirmed that the MoS_2_@CNFs@rGO has a voltage plateau at 1.4 V (vs. Na/Na^+^) and a slope thereafter. This can be explained by a reaction caused by the insertion of Na^+^ into MoS_2_ (Equation (4)) and a subsequent conversion reaction (Equation (5)). Sodium polysulfide intermediate (Na_2_S_x_, where x = 2 to 5) generated by the conversion reaction are easily dissolved in liquid electrolyte and move to the Na anode (“polysulfide shuttling”), leading to capacity loss and adverse effects on battery operation. The overall reaction can be represented as [37,38,39]:

(4) $${\mathrm{MoS}}_{2}+{x\;\mathrm{Na}}^{+}+{x\;e}^{-}\;\to {\mathrm{Na}}_{x}{\mathrm{MoS}}_{2}\;(x<2)$$

(5) $${\mathrm{Na}}_{x}{\mathrm{MoS}}_{2}+\left(4-x\right){\;\mathrm{Na}}^{+}\to \;2{\;\mathrm{Na}}_{2}S\;+\mathrm{Mo}$$

The voltage plateau generated at about 1.6 V in the charging process occurred due to the reduction of Na_2_S to S. After the initial cycle, the SIB cell containing the MoS_2_@CNFs@rGO electrode shows a highly reversible Na^+^ storage ability. We evaluated the long-term stability of MoS_2_@CNFs@rGO, as shown in Figure 6b. The MoS_2_@CNFs@rGO delivered a high discharge capacity of 345.8 mAh g^−1^ at the 90th cycle with a CE of 99.8%. In addition, the rate capability of MoS_2_@CNFs@rGO was tested at various current densities between 0.1~20 A g^−1^ (Figure 6c). At lower rates, the MoS_2_@CNFs@rGO exhibited similar capacity retention to that of MoS_2_@CNFs. On the other hand, the rate capability of MoS_2_@CNFs@rGO was gradually improved at higher rates (>0.5 A g^−1^). Moreover, the MoS_2_@CNFs@rGO outperformed the MoS_2_@CNFs even at a super-fast rate of 20 A g^−1^. This outstanding ability to store Na^+^ was also able to be used for Li^+^ storage (Figure S5).

All things taken together, we elucidated the reaction mechanism of MoS_2_@CNFs@rGO, based on the following factors: (I) MoS_2_ nanoflakes with enlarged interlayer spacing and short lateral distance between each flake are beneficial for storing Na^+^ in the CNFs. (II) The CNFs serve as a continuous passage for electrons and storage matrix for the active materials (Na^+^ and S). (III) The rGO is synergistically advantageous in terms of fast kinetics. Eventually, it turns out that the MoS_2_@CNFs@rGO synthesized by our fabrication approach enables the SIB cells to show remarkable performance toward Na^+^ insertion and conversion reactions.

## 4. Conclusions

In summary, we report a straightforward approach to fabricate a composite consisting of MoS_2_ nanoflakes confined in CNFs wrapped with a rGO net for SIB anodes. We effectively generated randomly distributed MoS_2_ nanoflakes (a few layers) in the CNFs via thermolysis and wrapped the rGO onto the MoS_2_@CNFs to enable the composite to be active for Na^+^ storage. The MoS_2_ nanoflakes possessing short lateral and expanded interlayer distances would be favorable for reversible insertion/desertion of Na^+^. In addition, the strongly interconnected CNFs and rGO net promoted electron transfer through the whole electrode, rendering a high rate of capability and cyclability. Furthermore, the rGO would preclude the dissolution of S stemming from the conversion reactions at the outermost surfaces of the composite materials. The MoS_2_@CNFs@rGO showed excellent cycle retention with a specific capacity of 345.8 mAh g^−1^ at a current density of 100 mA g^−1^ from the initial cycle to the 90th cycle. We elucidated that in the MoS_2_-based anodes undergoing conversion reactions, caging the active materials in the electronically conductive scaffold is critical for improving electrochemical battery performance.

## Figures and Tables

**Table 1:** Contents of the carbon and sulfur components of MoS_2_@CNFs and MoS_2_@CNFs@rGO.

Samples	Carbon, C (wt %)	Sulfur, S (wt %)
MoS_2_@CNFs	23.6	29.8
MoS_2_@CNFs@ rGO	34.3	20.9

## Data Availability

Data are contained within the article.

## Supplementary Materials

The following are available online at https://www.mdpi.com/article/10.3390/nano11102691/s1. Figure S1: EDS spectrum of rGO@MoS2@CNFs, Figure S2: XPS analysis for MoS2@CNFs@rGO and MoS2@CNFs and for (a) C1s and (b) Mo 3d, Figure S3: Raman analysis for (a) MoS2@CNFs@rGO and (b) MoS2@CNFs with classified peaks (I, IIII: sp3 bonding, II, IV: sp2 bonding), Figure S4: Charge–discharge curves of MoS2@CNFs at a current density of 100 mA g−1, Figure S5: Lithium-ion battery performance of MoS2@CNFs@rGO.
