# Supplementary material for: Reduced Graphene-Oxide-Encapsulated MoS2/Carbon Nanofiber Composite Electrode for High-Performance Na-Ion Batteries
Source: Nanomaterials (Basel). 2021 Oct 13;11(10):2691. doi: 10.3390/nano11102691 (PMC8539876; doi:10.3390/nano11102691)
Supplement: Supplementary file 1 [file nanomaterials-11-02691-s001.zip › nanomaterials-1401833-supplementary.pdf]

nanomaterials

Supporting Information

Reduced Graphene-Oxide-Encapsulated MoS_2/Carbon Nanofiber Composite Electrode for High-Performance Na-Ion Batteries

Su-Ho Cho [1,†], Jong-Heon Kim [2,†], Il-Gyu Kim [3], Jeong-Ho Park [3], Ji-Won Jung [3,*], Hyun-Suk Kim [2,*] and Il-Doo Kim [1,*]

1 Department of Materials Science and Engineering, Korea Advanced Institute of Science and Technology (KAIST), Daejeon 34141, Korea; caca1108@kaist.ac.kr
2 Department of Materials Science and Engineering, Chungnam National University, Daejeon 34134, Korea; mapig2@naver.com
3 School of Materials Science and Engineering, University of Ulsan, Ulsan 44776, Korea; ilgyu.kim.96@gmail.com (I.-G.K.); fjdk9936@naver.com (J.-H.P.)
* Correspondence: jwjung4@ulsan.ac.kr (J.-W.J.); khs3297@cnu.ac.kr (H.-S.K.); idkim@kaist.ac.kr (I.-D.K.)
† S.-H.C. and J.-H.K. contributed equally to this work.

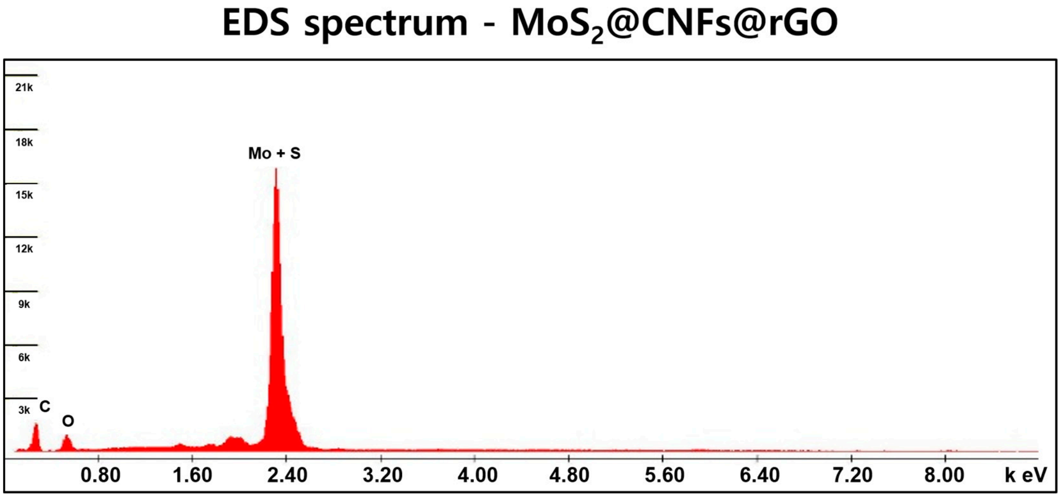

Figure S1. EDS spectrum of rGO@MoS_2@CNFs.

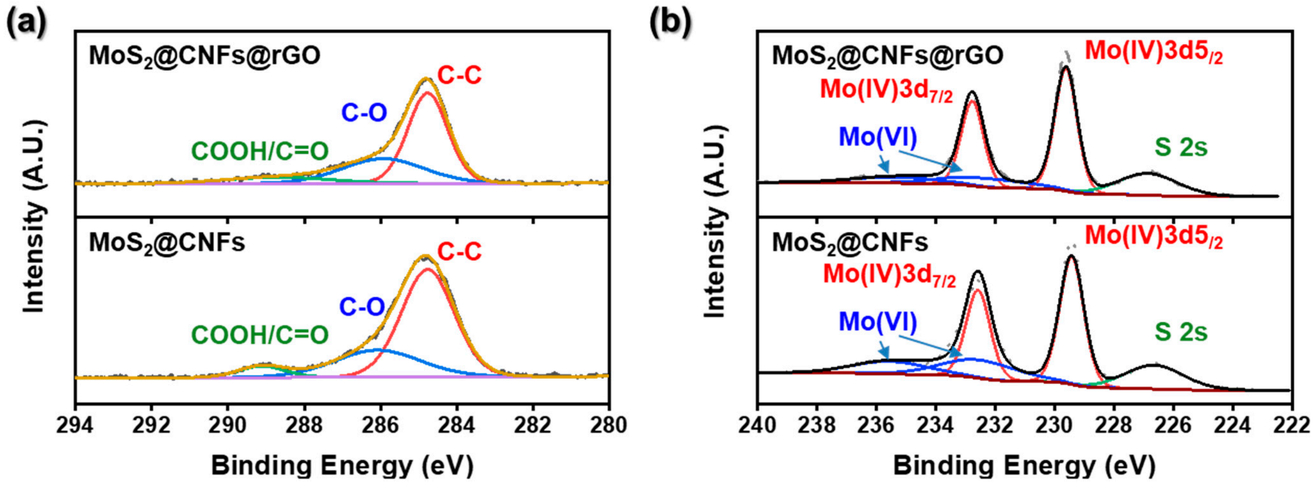

Figure S2. XPS analysis for MoS_2@CNFs@rGO and MoS_2@CNFs and for (a) C1s and (b) Mo 3d.

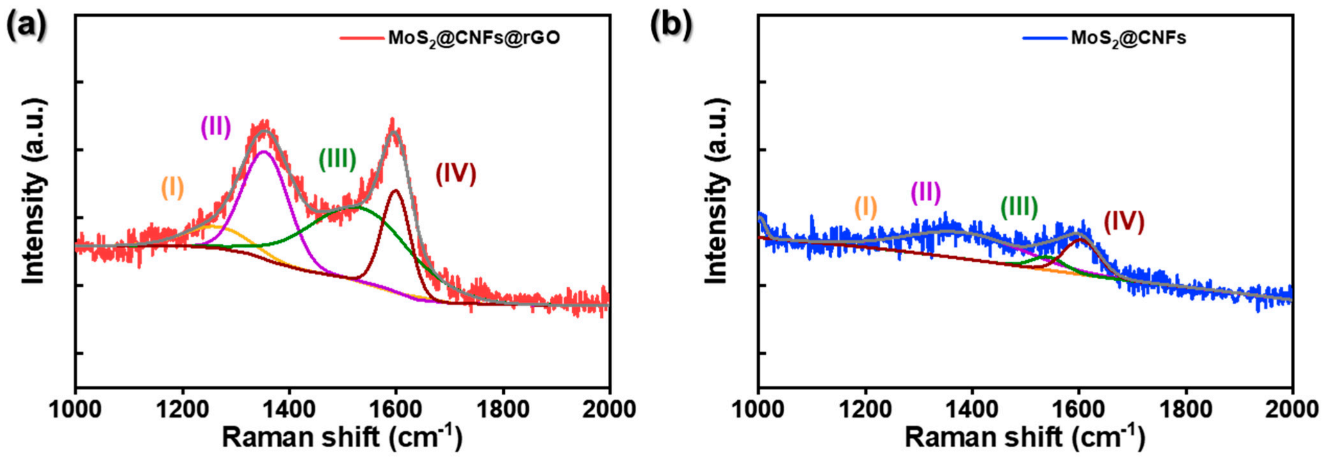

Figure S3. Raman analysis for (a) MoS_2@CNFs@rGO and (b) MoS_2@CNFs with classified peaks (I, IIII: sp^3 bonding, II, IV: sp^2 bonding).

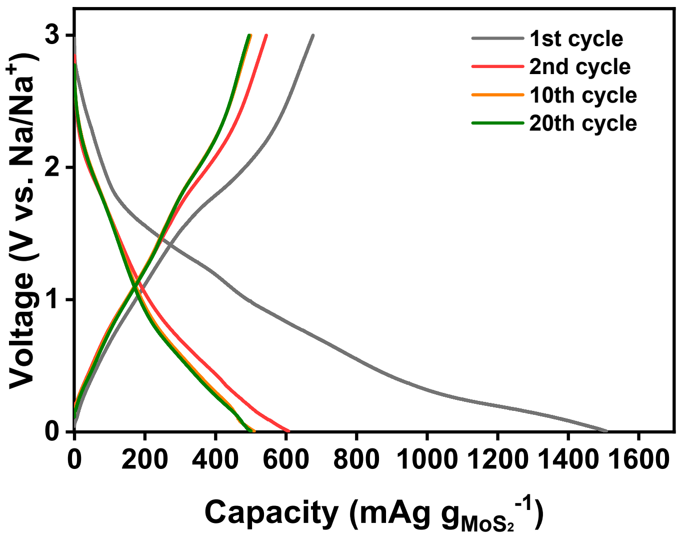

Figure S4. Charge-discharge curves of MoS_2@CNFs at a current density of 100 mA $g^{-1.}$

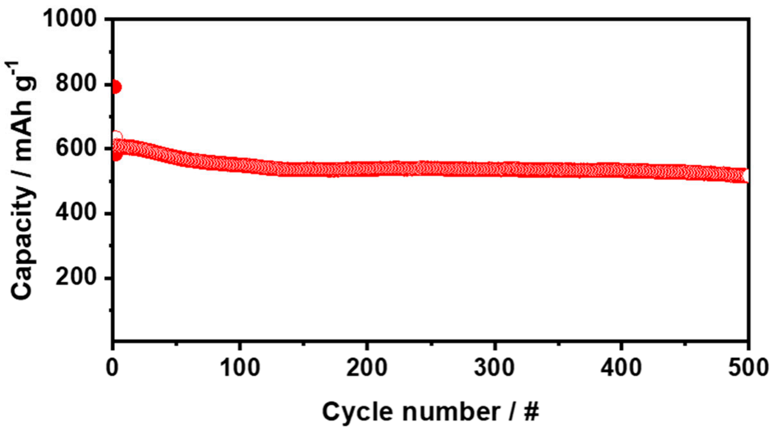

Figure S5. Lithium ion battery performance of MoS_2@CNFs@rGO.
